# Uncertainty avoidance and investment underdiversification

**DOI:** 10.1371/journal.pone.0272222

**Published:** 2022-08-09

**Authors:** Xinmeng Tang, Xiaoguang Zhou

**Affiliations:** School of Economics and Management, University of Science and Technology Beijing, Beijing, People’s Republic of China; University of Brescia: Universita degli Studi di Brescia, ITALY

## Abstract

The relationship between the cultural dimension of uncertainty avoidance and investment underdiversification is examined. A theoretical link between uncertainty avoidance and ambiguity is established, that is, cultural uncertainty avoidance captures the aversion attitude towards ambiguity at the national group level, thus, cultural dimension of uncertainty avoidance influences investors’ behavioral biases of home bias and the investment abroad concentration. The empirical results show that investment underdiversification is significantly and positively affected by the degree of uncertainty avoidance and robustness tests support this conclusion. A further analysis reveals that uncertainty avoidance moderates the effects of ambiguity on investment underdiversification, whereas the effects of uncertainty avoidance are mediated by the status quo bias.

## 1. Introduction

Modern Portfolio Theory suggests that adopting a diversification strategy is essential for minimizing idiosyncratic risks [1]. Numerous empirical studies have nevertheless shown that the diversification degree of investors’ portfolios is not sufficient to eliminate these risks. [2] found that out of 17056 US investors, 34.1% hold only one stock, 50% hold less than two stocks, and only 10.7% hold more than ten stocks. [3] determined that the investor’s portfolio contains on average only 3.14 stocks, and more than 70% of portfolios are insufficiently diversified.

Insufficient diversification, underdiversification, can also be observed in the portfolios of investors who pursue domestic as well as cross-border investments. The tendency of investors to allocate assets predominantly in domestic markets instead of making globally diversified investments has been termed in financial research as the home bias. [4] noted that US, Japanese and British investors clearly prefer to invest in domestic markets, as the proportion of their equity allocated to domestic stocks was 94%, 98%, and 82%, respectively. In addition, cross-border investments may also exhibit underdiversification when assets held in foreign equity are not sufficiently diversified among foreign markets.

This paper investigates the effects of investors’ behavioral biases from the perspective of overinvestment in a home country and overconcentration of investments abroad. The Home Bias index can effectively capture the degree of underdiversification caused by investors’ propensity to prioritize investments in domestic markets, but it fails to reflect the degree of diversification of investors’ investments abroad. Although underdiversification of investments abroad is a common phenomenon, it has not yet received adequate academic attention. Therefore, this paper constructs the Investment Abroad Concentration index that can estimate the concentration of investments abroad. As this index is created based on the Theil Entropy index that can capture deviations from a perfectly equal distribution [5, 6], a portfolio is considered insufficiently diversified when investors concentrate their investments abroad in only a few foreign markets.

In recent years, with the introduction of the national cultural dimension theory [7], an increasing number of scholars have begun to explore the significant factors of national culture or explain such behavioral bias phenomenon. National culture, as a soft non-institutional force, has a subtle impact on financial decision-making by affecting individual financial behaviors, habits, preferences, and choices, which is confirmed by a large number of empirical studies [8–13].

Among all cultural dimensions, uncertainty avoidance could capture the degree to which people are uncomfortable with unknown or uncertain [7, 14]. Research on culture at the national level has consistently shown it to relate positively with anxiety and ambiguity though there are also cultural implications of the relationship between role ambiguity and strains at the individual level [15–17]. While plenty of research confirmed the role of ambiguity aversion psychological characteristics, ambiguity-averse investors would allocate more portfolio wealth to the home country and ceteris paribus less to foreign assets [18]. However, at the national group level, there is a lack of literature on the interaction between uncertainty avoidance and investment underdiversification.

Based on the above analysis, using annual data on 51 economies that cover the period from 2001 to 2018, this study aims to explore the effects of uncertainty avoidance on investment underdiversification. Data on uncertainty avoidance were obtained from the 2015’s edition of Hofstede’s cross-cultural survey, and data on variables used to construct underdiversification indexes were taken from the International Monetary Fund’s Coordinated Portfolio Investment Survey (CPIS). A set of economic and financial control variables is introduced into the model, since it has been confirmed that the formal institutions may significantly influence investors’ behavioral biases. The empirical results reveal that uncertainty avoidance is a significant explanatory variable of investment underdiversification. When uncertainty avoidance increases by one standard deviation, then the degrees of home bias and the concentration of investments abroad increase on average by 6.42 and 0.07 units, respectively. The robustness of these results is confirmed by employing alternative variables and testing for endogeneity with the instrumental variable approach.

This paper also investigates whether investment underdiversification is affected by ambiguity, namely political, economic, and legal ambiguity. The results reveal that when political, economic, and legal ambiguity increase by one standard deviation, then the degrees of home bias (concentration of investment abroad) increase on average by 10.80 (0.01), 5.82 (0.03), and 16.61 (0.05) units, respectively. Moreover, further analysis shows that the effects of ambiguity on investment underdiversification are positively moderated by uncertainty avoidance, and the effect of uncertainty avoidance on investment underdiversification is mediated by the investors’ status quo bias.

Two outstanding studies examining investment underdiversification share certain common features with this paper. [19] investigated the investment bias of the mutual fund from 26 countries investing in 48 countries and found that the cultural dimensions of uncertainty avoidance, individualism, and cultural distance all play a significant role in determining asset allocation. [20] used the data on 37,000 investment institutions from over 60 countries investing in more than 80 countries and discovered that the cultural dimensions of uncertainty avoidance, individualism, masculinity, long-term orientation, and cultural distance provide additional insight into institutional investors’ decisions on asset allocation. Moreover, [20] also demonstrated that culture impacts investors’ behavior directly and not merely through indirect channels such as legal and regulatory frameworks.

Great differences can be found between these two studies and this paper, which are reflected in the main contributions of this paper. First, the Investment Abroad Concentration (*IAC*) index is constructed to capture the underdiversification degree of assets invested abroad. Although this index is certainly similar to the foreigner diversification index created by [20], the Investment Abroad Concentration (*IAC*) index can better reflect the investment underdiversification at the country level, whereas the foreign diversification index is more suitable for measuring the underdiversification degree of investments made by institutional investors such as mutual funds. Second, the theoretical explanation of the effects of uncertainty avoidance on investment underdiversification is deepened. Extant research has proved that culture plays an important role in investment underdiversification, however, no research has yet provided a solid theoretical interpretation of the high explanatory power of culture. This paper shows that uncertainty avoidance is an important factor influencing investment underdiversification of investments, as this cultural dimension reflects the investors’ ambiguity aversion, which affects. Third, the internal mechanism of the relationship between uncertainty avoidance and investment underdiversification is explored. This paper discovers that uncertainty avoidance moderates the effect of ambiguity on investment underdiversification, and investors’ status quo bias mediated the effect of ambiguity on investment underdiversification. Fourth, extant researchers perform empirical analysis using mutual fund data, while our analysis focuses on equity underdiversification using the data from CPIS because of the particularity of investors’ behaviors in equity investment. According to [21], more home bias behaviors can be found in equity markets because of high expected returns in equity markets (normally negative-skewed distribution).

The rest of the paper is organized as follows: The "Theoretical frameworks" section presents the index construction and hypothesis development. The "Sample selection and construction of variables" section talks about the sample selection and variables construction. Results and discussions of uncertainty avoidance and underdiversification are shown in the "Empirical results and discussions" section. The "Conclusions and implications" section shows the conclusions and practical implications of this research.

## 2. Theoretical frameworks

### 2.1 Investment underdiversification

#### 2.1.1 Home bias

The investor’s preference for investing in domestic markets is measured by the Home Bias (*HB*) index, that has proven to be a reliable method of measuring the rate of overinvestment in a home country [22]. Home Bias (*HB*) index calculation is described by Eq (1).

$$HB_{i,t}=\frac{w_{i,t}^{*}‐x_{i,t}^{f}}{w_{i,t}^{*}},$$ (1)

where *HB*_*i*,*t*_ represents the degree of home bias of country *i* at time *t*, *x*^*f*^_*i*,*t*_ denotes the proportion of assets invested abroad to the total investment assets of country *i* at time *t*, and w^*^_i,*t*_ is the weight of market capitalization of country *i* in the global market capitalization at time *t*.

#### 2.1.2 Investment abroad concentration

To assess the underdiversification of investments abroad, this paper constructs Investment Abroad Concentration (*IAC*) index based on the Theil index. [23] originally proposed this method as a way to measure income distribution, but since this entropy index is able to capture even small deviations from perfect equality, it has become widely used in research on cross-border differences in income inequality [24], innovation ability and productivity [25]. In addition, it has also recently been used to measure the concentration of foreign direct investment [5, 6]. The Investment Abroad Concentration (*IAC*) index calculation is described by Eq (2).

$$IAC_{i,t}=\frac{1}{\log_{2}N}\sum_{l=1}^{N}\left(x_{i,t}^{l}\cdot \log_{2}x_{i,t}^{l}\right),$$ (2)

where *IAC*_*i*,*t*_ represents the degree of concentration of investments abroad of country *i* at time *t*, *N* is the total number of countries in which a country *i* invested at time *t*, and *x*^*l*^_*i*,*t*_ denotes the proportion of county *i*’s investment assets invested in country *l* (*l* = 1, 2, 3,…, *N*) to the total country *i*’s assets invested abroad at time *t*, *x*^*1*^_*i*,*t*_*+x*^*2*^_*i*,*t*_ +…+*x*^*N*^_*i*,*t*_ = 1. The value rate of *IAC*_*i*,*t*_ is (0,1), when investors distribute their assets invested abroad among many foreign markets (*N*→∞, *x*^*l*^_*i*,*t*_→0), then *IAC*_*i*,*t*_ approaches 0, and the portfolio is well diversified. Analogously, when investors concentrate their assets invested abroad in a certain market (*N* = 1, *x*^*l*^_*i*,*t*_ = 1), then *IAC*_*i*,*t*_ approaches 1, and the portfolio is underdiversified.

### 2.2 Culture of uncertainty avoidance

Uncertainty avoidance is related to the unpredictability of the future, reflecting the degree to which people are uncomfortable with the uncertainty or the unknown [7, 14]. It reflects the extent to which a culture educates its people to feel uncomfortable or comfortable in unstructured conditions. Unstructured circumstances are novel, unfamiliar, shocking, or out of the ordinary [26, 27]. Uncertainty avoidance culture aims to reduce the potential of such circumstances by tight laws and standards, as well as safety and security measures, while countries with high uncertainty avoidance are also more emotional and motivated by inner nervous energy [28].

Research on culture at the national level has consistently shown it to relate positively with anxiety and ambiguity though there are also cultural implications of the relationship between role ambiguity and strains at the individual level [15–17]. [7, 29, 30] theorized that culture can reflect the common psychological reactions of its members at the national level, and affects individual mental programs, which condition individuals’ responses to their environment. Further, ambiguity refers to situations where probabilities are unknown [18]. Moreover, as Hofstede explained, ambiguity aversion is a component of [7, 14] uncertainty avoidance dimension, which captures a society’s tolerance for uncertainty and ambiguity. The national culture of uncertainty avoidance thereby can capture the ambiguity aversion psychological reactions at the group level [17, 31].

### 2.3 Hypothesis

#### 2.3.1 Uncertainty avoidance and investment underdiversification

The culture of uncertainty avoidance could explain home bias underdiversification when uncertainty avoidance is poxy of the individual’s ambiguity aversion [32–34]. A prominent explanation in the literature on equity home bias is ambiguity aversion [32, 33, 35, 36]. A long strand of the literature starting with Ellsberg (1961) [37] has shown that subjects are averse to ambiguity [38]; however, relative to the domestic stock market, foreign stocks are relatively ambiguous, and own-company stock is relatively unambiguous. More specific explanations lie in that ambiguity-averse investors shy away from ambiguity and allocate their portfolio wealth to assets with less ambiguity, the resulting portfolio deviates from a traditional mean-variance portfolio allocation [33, 39, 40]. Similarly, the degree of ambiguity would decrease while the level of familiarity towards securities increase, when fixed asset are allocated to more markets (abroad concentration declining) for ambiguity-averse investors. Then given that the cultural dimension of uncertainty avoidance can reflect the investors’ attitude towards ambiguity, Hypotheses 1 and 2 are proposed.

**H1: Uncertainty avoidance is positively correlated to investors’ home bias.**
**H2: Uncertainty avoidance is positively correlated to the concentration of investments abroad.**

#### 2.3.2 Internal mechanism of uncertainty avoidance and investment underdiversification

Ambiguity towards securities or markets is one of the factors causing the cross-border investment bias [18, 33, 40]. As proved by [18], an increase in the level of domestic ambiguity relative to the level of ambiguity would increase investors’ foreign holdings; contrarily, an increase in the level of foreign ambiguity relative to the level of domestic ambiguity leads to a decrease in their foreign holdings. To sum up, the relative ambiguity of the assets of the aboard deteriorates, ambiguity averse investors would allocate more portfolio wealth to the home country and ceteris paribus less to foreign assets [18], as a result, home bias degree increases. Thereby, Hypothesis 3 thereby is proposed.

Different degrees of investors’ ambiguity aversion psychological traits would modify investors’ biased behaviors towards different ambiguity [18, 33, 34, 40]. [18] confirmed the moderator effect of ambiguity aversion, after an increase in the level of foreign ambiguity relative to the level of domestic ambiguity, more ambiguity averse investors decrease their foreign holdings by a larger amount than less ambiguity averse investors, leading to a relative increase in foreign bias [41, 42]. Then given that the cultural dimension of uncertainty avoidance can reflect the investors’ attitude towards ambiguity; therefore, Hypothesis 4 is proposed.

**H3: Ambiguity is positively correlated to investment underdiversification.**
**H4: Uncertainty avoidance moderates the effect of ambiguity on investment underdiversification.**

A substantial body of recent studies suggests that individual decision-makers frequently place an extra value on their default alternatives or status quo choice, a phenomenon known as the status quo bias. The notion that the case of uncertainty or ambiguity might be of special relevance for the study of status quo bias is not new. The existence of uncertainty may cause the agent to become "confused," causing her preference relation to becoming incomplete; then, the agent maintains her status quo unless she finds something better based on her (incomplete) preference, a presumption very similar to the status quo bias [43, 44].

Furthermore, it is plausible to include the status-quo bias as a possible cause of non-optimal diversification [43–46]. In situations when investors accede to an existing security portfolio, they often tend to postpone or even completely fail to adjust the portfolio structure. Even if the differing performances of the stocks in the portfolio result in an unintentional imbalance, many investors hesitate to take necessary action because of fear of changing the portfolio. Aside from fear, this lack of action is frequently motivated by a refusal to accept responsibility for the portfolio’s future profit, or lack thereof. Many investors are afraid of regretting their own actions [47–49]. Hypothesis 5 thus is proposed.

**H5: Investors’ status quo bias mediates the effect of uncertainty avoidance on investment underdiversification.**

## 3. Sample selection and construction of variables

### 3.1 Sample selection

The research sample consists of 51 economies, including countries in the Americas, Europe, Africa, Asian Pacific, and the Middle East. As investments abroad made by investors from these countries account for more than 90% of global investments abroad, the research sample can be considered highly representative. The sample countries were selected using the following steps. First, this paper obtained the data from the International Monetary Fund’s Coordinated Portfolio Investment Survey (CPIS) survey, which focused on foreign equity investment in 67 countries around the world. Second, after assessing Hofstede’s data on national uncertainty avoidance, the sample had to be reduced to 57 countries. Third, due to insufficient data on investments abroad, Latvia, Estonia, and Uruguay were removed from the sample. Finally, Ireland, Luxembourg, and Panama were also discarded from the sample as the data on their total investments were unavailable. Thus, this paper uses annual data and covers the period from 2001 to 2018 in 51 economies, and the year 2001 is selected as the beginning of the study period because investments abroad began to flourish in this century.

### 3.2 Construction of variables

#### 3.2.1 Investment underdiversification variables

The specific calculations of investment underdiversification variables (*UNDV*) are described by Eqs (1) and (2) and the data used for calculation originate from International Monetary Fund’s CPIS. Table 1 shows descriptive statistics for investment underdiversification variables. The codes of investigated countries are reported in the 1st (6th) column, the mean and standard deviation of the Home Bias (*HB*) index are presented in the 2nd (7th) and 3rd (8th) columns, respectively, and the mean and standard deviation of Investment Abroad Concentration (*IAC*) index are shown in the 4th (9th) and 5th (10th) columns, respectively. The Netherlands (NLD) has the lowest degree of home bias, which is typical for an export-oriented economy, and the highest degree of home bias can be found in several Asian and Latin American countries. In some countries, the value of the Investment Abroad Concentration (*IAC*) index varies significantly from the value of the Home Bias (*HB*) index. For example, Argentina (ARG) and Venezuela (VEN) show a high tendency to invest in domestic markets, but a low tendency to concentrate their investments abroad, confirming that Home Bias (*HB*) and Investment Abroad Concentration (*IAC*) indexes capture different aspects of investors’ behavior affecting investment underdiversification. In addition, the degree of home bias calculated using the national data is higher than the degree of home bias of institutional investors presented in [19, 20], which is consistent with the argument that, compared to institutional investors, individual investors are more susceptible to irrational factors and therefore exhibit a greater behavioral bias.

**Table 1:** Mean and standard deviation of *HB* and *IAC* by countries.

code	home bias (*HB*)		investment abroad concentration (*IAC*)		code	home bias (*HB*)		investment abroad concentration (*IAC*)	
	AVG. (%)	SD.	AVG.	SD.		AVG. (%)	SD.	AVG.	SD.
ARG	79.03	0.04	0.15	0.08	MYS	93.52	0.05	0.60	0.09
AUS	75.93	0.07	0.55	0.04	MLT	60.12	0.57	0.63	0.16
AUT	44.88	0.09	0.63	0.01	MEX	94.65	0.04	0.31	0.05
BGD	99.99	0.00	0.71	0.08	NLD	32.45	0.17	0.58	0.01
BEL	44.59	0.10	0.54	0.02	NZL	52.86	0.09	0.53	0.16
BRA	97.95	0.02	0.53	0.04	NOR	37.10	0.21	0.62	0.02
BGR	88.39	0.29	0.67	0.10	PAK	99.61	0.01	0.53	0.19
CAN	68.14	0.11	0.44	0.03	PER	79.08	0.02	0.47	0.02
CHL	73.12	0.14	0.43	0.07	PHL	99.68	0.01	0.59	0.16
HKG	80.52	0.05	0.58	0.09	POL	93.60	0.05	0.67	0.07
CHN	96.56	0.01	0.47	0.01	PRT	52.62	0.21	0.67	0.05
COL	92.05	0.07	0.37	0.05	ROU	97.02	0.03	0.64	0.16
CRI	90.27	0.09	0.58	0.09	RUS	99.17	0.00	0.52	0.07
CZE	73.36	0.22	0.67	0.04	SGP	56.96	0.07	0.70	0.04
DNK	71.27	0.19	0.60	0.02	SVK	78.62	0.09	0.68	0.04
FIN	56.86	0.25	0.69	0.06	SVN	59.53	0.09	0.68	0.02
FRA	63.45	0.06	0.59	0.01	ZAF	86.22	0.03	0.42	0.05
DEU	51.35	0.14	0.61	0.01	ESP	80.81	0.11	0.65	0.06
GRC	82.71	0.10	0.53	0.10	SWE	58.33	0.02	0.58	0.03
HUN	70.64	0.14	0.60	0.08	CHE	54.63	0.05	0.58	0.01
IND	99.89	0.01	0.52	0.10	THA	97.47	0.02	0.61	0.11
IDN	94.54	0.20	0.57	0.11	TUR	99.82	0.00	0.52	0.06
ISR	76.19	0.20	0.38	0.02	GBR	59.97	0.07	0.62	0.04
ITA	57.23	0.04	0.51	0.05	USA	74.56	0.06	0.66	0.02
JPN	81.16	0.08	0.53	0.03	VEN	99.52	0.01	0.34	0.14
KOR	90.60	0.06	0.55	0.04					

Furthermore, this paper constructs an alternative index of home bias *AL_HB*_*i*,*t*_ and an alternative index of investments abroad concentration *AL_IAC*_*i*,*t*_ to confirm the robustness of the results. *AL_HB*_*i*,*t*_ index is obtained by Eq (1), but CPIS data for calculating the weight of a country’s market capitalization in the global market capitalization are replaced with data from World Bank Open Database, and *AL_IAC*_*i*,*t*_ index is obtained by Eq (2), but the number of foreign countries in which a country invests is excluded, as shown in Eq (3).


$$AL\_IAC_{i,t}=\sum_{l=1}^{N}\left(x_{i,t}^{l}\cdot \log_{2}x_{i,t}^{l}\right).$$ (3)

#### 3.2.2 Uncertainty avoidance variables

Data on the cultural dimension of uncertainty avoidance (*UAI*) were obtained from Hofstede’s cross-culture survey. Hofstede and his team have been irregularly updating the culture score data of different countries on their website since 2008. Uncertainty avoidance data used in this paper were taken from the latest publication in 2015.

Culture is a national attribute formed in the long-term history and retained in real life. It is generally believed that culture is relatively stable and does not change in a short period of time, and thus can be regarded as given [50]. Thus, this paper assumes uncertainty avoidance variables to be time-constant.

Furthermore, this paper employs three alternative indexes of uncertainty avoidance to confirm the robustness of the results. The first alternative index is *TK_UAI* created by [51], which provided a framework to update Hofstede’s indexes based on the changing economic environments within countries. The second alternative index is compiled by the Global Learning and Observation to Benefit Environment (GLOBE) project, which extended Hofstede’s concept of five cultural dimensions to nine dimensions and thus provided a more comprehensive description of cultural attributes [52]. Thirdly, several researchers try to identify and measure the level of ambiguity aversion at the whole country level [18, 36, 53], the calculated national wide ambiguity aversion index in the study of [53] is used as the last alternative index.

#### 3.2.3 Ambiguity variables

Scholars have not reached an agreement on how to measure the degree of ambiguity faced by cross-border investors [18, 40]. While politics, economics, and regulation are three main macroscopic factors when performing transnational investment. If the home country has relatively high values in these three aspects, then the ambiguity associated with investing in the domestic market is low, and to some extent, the ambiguity of cross-border investments decreases [18]. Specifically, ambiguity (*AB*) is proxied by three variables, the degree of political stability (*PS*), economic freedom (*EF*), and the rule of law (*RL*). Political stability (*PS*) reflects the extent to which the political situation in the home country can be considered stable. Economic freedom (*EF*) represents the extent to which individuals are free to work, produce, consume and invest. The rule of law (*RL*) captures the perception of the extent to which individuals have confidence in and abide by the rules of society. The data on political stability and the rule of law was taken from the Worldwide Governance Indicators (WGI) project, and data on economic freedom originate from the Heritage Foundation (HF).

#### 3.2.4 Investors’ status quo bias variables

The status quo bias is obtained using the method introduced in [54]. This method assumes that the investment underdiversification in the current year depends on the degree of investment underdiversification in the previous year. Therefore, this paper constructs a varying-coefficient model with underdiversification in year *t* (proxied by *HB*_*i*,*t*_ and *IAC*_*i*,*t*_) as explained variables and the underdiversification in year *t*-1 (proxied by *HB*_*i*,*t-*1_ and *IAC*_*i*,*t-*1_) as explanatory variables. A regression of panel data is performed in order to obtain the regression coefficient of each country. The degree of status quo bias of country *i* (denoted as *HB_SQB*_*i*_ and *IAC_SQB*_*i*_) is then measured by the regression coefficient of that country.

#### 3.2.5 Other control variables

This paper also considers control variables from macroeconomic and macro-financial perspectives. Macroeconomic conditions are represented by GDP per capita and GDP growth rate, whereas the ratio of stock traded to GDP, the ratio of the market capitalization of listed companies to GDP, the ratio of domestic credit provided by the financial sector to GDP, and market capitalization of listed companies reflect macro-financial development. Data on control variables were obtained from World Bank Open Database.

This paper predicts that the macroeconomic and macro-financial conditions of home countries are negatively correlated to the concentration of investments abroad, as home countries with a higher economic level can provide investors with greater financial support to expand investment abroad, and home countries with a higher degree of macro-financial development can offer investors more complete and reliable channels for investing abroad as well as more accurate information on investments in foreign markets.

The relationship between control variables and home bias is, however, more difficult to predict. On the one hand, as with the concentration of investments abroad, a higher level of economic and financial development can provide better financial support and accurate information on overseas investments, and therefore a negative correlation may occur. On the other hand, a higher level of economic and financial development can also offer more domestic investment opportunities, and therefore a positive correlation may occur. Extant research reflects this diverse relationship. [19] proved that home bias is positively correlated with the level of financial development proxied by the ratio of the market capitalization of listed companies to GDP in developed markets, but this correlation is negative in emerging markets. [20] reached a similar conclusion when analyzing the relationship between investors’ home bias and economic conditions represented by GDP per capita. However, the above research was based on mutual funds data and thus described the behavior of institutional investors, whereas this paper uses overall national data and therefore captures the behavior of all investors, both institutional and individual ones.

The specific descriptions and data sources of the explanatory, explained, and control variables are shown in Table 2.

**Table 2:** Summary descriptions and data sources of variables.

Variable	Variable descriptions	Sources
**Explanatory variables:**		
*UAI*_*i*_	Uncertainty avoidance degree of country *i*	Hofstede cultural data
*TK_UAI*_*i*_	Alternative index of uncertainty avoidance degree of country *i*	Tang and Koveos (2008)
*GLOBE_UAI*_*i*_	Alternative index of uncertainty avoidance degree of country *i*	House et al. (2004)
*AA_UAI*_*i*_	Alternative index of uncertainty avoidance degree of country *i*	Rieger et al. (2015)
**Explained variables:**		
*HB*_*i*,*t*_	Home bias degree of country *i* in year *t*	CPIS
*AL_HB*_*i*,*t*_	Alternative index of home bias degree of country *i* in year *t*	CPIS and World Bank Open Data
*IAC*_*i*,*t*_	Investment abroad concentration degree of country *i* in year *t*	CPIS
*AL_IAC*_*i*,*t*_	Alternative index of investment abroad concentration degree of country *i* in year *t*	CPIS
**Ambiguity variables:**		
*PS*_*i*,*t*_	Political stability degree of country *i* in year *t*	WGI
*EF*_*i*,*t*_	Economic freedom degree of country *i* in year *t*	HF
*RL*_*i*,*t*_	The rule of law degree of country *i* in year *t*	WGI
**Investors’ status quo bias variables:**		
*HB_SQB*_*i*_	Investors’ status quo bias degree based on the home bias of the country *i*	
*IAC_SQB*_*i*_	Investors’ status quo bias degree based on investment abroad concentration of country i	
**Control variables:**		
*perGDP*_*i*,*t*_	GDP per capita of country *i* in year *t*	World Bank
*GDP growth*_*i*,*t*_	GDP growth rate of country *i* in year *t*	World Bank
*(ST / GDP)*_*i*,*t*_	The ratio of stock traded to GDP of country *i* in year *t*	World Bank
*(MC / GDP)*_*i*,*t*_	The ratio of the market capitalization of listed companies to GDP of country *i* in year *t*	World Bank
*(FC / GDP)*_*i*,*t*_	The ratio of domestic credit provided by the financial sector to GDP ‎of country *i* in year *t*	World Bank
*MC*_*i*,*t*_	The market capitalization of listed companies of country *i* in year *t*	World Bank

## 4. Empirical results and discussions

### 4.1 Uncertainty avoidance and investment underdiversification

#### 4.1.1 Basic model

To test Hypotheses 1 and 2, this paper constructs the Uncertainty Avoidance-Underdiversification (*UAI*- *UNDV*) model that can be expressed by Eq (4).

$$\begin{array}{l} UNDV_{i,t}=\beta_{0}+\beta_{1}UAI_{i}+\beta_{2}perGDP_{i,t}+\beta_{3}GDP\;growth_{i,t}+\beta_{4}\ln{\left(ST/GDP\right)}_{i,t}+\beta_{5}\ln{\left(MC/GDP\right)}_{i,t}\\ \;+\beta_{6}\ln{\left(FC/GDP\right)}_{i,t}+\beta_{7}MC_{i,t}+u_{i,t}, \end{array}$$ (4)

where *UNDV*_*i*,*t*_ denotes the investment underdiversification degree of country *i* in year *t*, including *HB*_*i*,*t*_ and *IAC*_*i*,*t*_, *β*_*m*_ denotes the coefficient of the *m*th variables (*m* = 1, 2, …, 7), *u*_*i*,*t*_ denotes the model residuals.

To avoid a spurious regression, the Levin-Lin-Chu (LLC), Breitung, Im-Pesaran-Shin (IPS), and Phillips-Perron (PP)-Fisher tests were used to test the stationarity of variables, and the results are reported in Table 3. *T* denotes the stationarity tests for the series with trend term, *I* denotes the stationarity tests for the series with intercept term, and *N* denotes the stationarity tests for the series without intercept or trend term.

**Table 3:** The results of stationarity tests.

Variable	Test	LLC	Breitung	IPS	Fisher-PP	Conclusion
*HB*	N	-5.36590***			127.498***	stationary
	I	-5.28217***		-3.23135***	112.797***	
	I and T	-3.06865***	1.45037*	-0.06059	70.1786	
*IAC*	N	1.90116			79.7951	stationary
	I	-7.75731***		-4.57993***	213.993***	
	I and T	-7.88890***	0.57251	-2.65123***	147.793***	
*perGDP*	N	8.09777			6.76667	stationary
	I	1.82323		3.49864	45.7481	
	I and T	-2.76266***	-0.84373	0.34670	86.4598*	
*GDP growth*	N	-4.02521***			147.013***	stationary
	I	-8.59378***		-7.02144***	169.896***	
	I and T	-9.16750***	-6.09870	-4.50841***	128.736***	
*ST/GDP*	N	4.45517			23.2008	stationary
	I	-3.93353***		0.77027	66.3158	
	I and T	-1.76367**	1.50989*	2.02927	55.4534	
*MC/GDP*	N	-0.48234			47.4195	stationary
	I	-13.6356***		-9.31368***	154.454***	
	I and T	-14.6785***	-5.01098	-7.64571***	120.498***	
*FC/GDP*	N	0.03308			33.9906	stationary
	I	-144.507***		-30.2179***	135.872***	
	I and T	-116.600***	-3.28299	-24.1847***	116.182***	
*MC*	N	4.01207			18.5619	stationary
	I	-3.55727***		-0.46283	73.9145	
	I and T	-6.53602***	-0.44249	-1.32854*	75.5458	

Note

*, **, *** indicate significance at the 10%, 5% and 1% levels, respectively.

As can be seen in Table 3, all variables are stationary and suitable for further regression analysis. Given that the focal cultural variables of this paper are time-constant, the fixed-effect analysis is not applicable [55, 56], and the unbalanced panel data used in this paper invalidates the two-way random effect. Thus, the Generalized Least Squares (GLS) regression method is employed due to its ability to effectively eliminate the heteroscedasticity and autocorrelation problems of models [57]. Furthermore, since the research sample time span *T* is 18 and the observation number *N* is 51, the structure of the sample may cause the heteroscedasticity problem in some cross-section observations. Thus, the cross-section weights and the Panel Correction Standard Error (PCSE) method are employed to correct the results. The regression results of Eq (4) (*UAI*- *UNDV* model) are presented in Table 4, where Models 1–5 aim to reflect the effects of uncertainty avoidance on portfolio underdiversification from the perspective of overinvestment in a home country, and Models 6–10 seek to reveal the effects of uncertainty avoidance on portfolio underdiversification from the perspective of overconcentration of investments abroad. All models consider the cultural variable of uncertainty avoidance with the exemption of Models 1 and 6. Whereas Models 2 and 7 use the variable of cultural uncertainty avoidance, Models 3 and 6 employ the alternative indexes. Models 4 and 9 are obtained by removing the United States from the sample. Models 5 and 10 use the sample that does not contain China.

**Table 4:** The regression results of the *UAI-UNDV* model.

**Panel A: Determinants of home bias (*HB*):**					
	**(1)**	**(2)**	**(3)**	**(4)**	**(5)**
	***HB***	***HB***	***AL_HB***	**ex. USA**	**ex. CHN**
*UAI*		0.2764 (5.7375)***	0.2744 (5.7341)***	0.3451 (6.7558)***	0.2764 (5.7375)***
*perGDP*	1.87E-07 (7.2969)***	3.51E-07 (7.0275)***	3.51E-07 (7.0775)***	4.15E-07 (7.6444)***	3.51E-07 (7.0275)***
*GDP growth*	0.4684 (2.3182)**	0.2868 (1.7505)*	0.2815 (1.7301)*	0.2048 (1.1859)	0.2868 (1.7505)*
*ln (ST / GDP)*	2.5454 (4.4915)***	4.2345 (5.9908)***	4.2846 (6.1136)***	4.4421 (5.6795)***	4.2345 (5.9908)***
*ln (MC / GDP)*	-0.9773 (-0.9303)	1.6942 (1.5059)	1.5347 (1.3780)	1.3212 (1.0670)	1.6942 (1.5059)
*ln (FC / GDP)*	-7.1834 (-6.5899)***	-13.2073 (-8.8918)***	-13.0261 (-8.8033)***	-12.4931 (-7.8249)***	-13.2073 (-8.8918)***
*MC*	-5.93E-13 (-7.2185)***	-2.80E-13 (-2.5117)**	-1.35E-13 (-1.2507)	-1.46E-12 (-1.9370)*	-2.80E-13 (-2.5117)**
*constant*	115.2282 (30.6328)***	106.2701 (21.239)***	106.1839 (21.4028)***	99.1006 (18.3080)***	106.2701 (21.239)***
Adjusted *R*-squared	0.7522	0.6305	0.5956	0.5403	0.6305
S.E. of regression	15.2882	14.5470	14.4401	15.2219	14.5470
*F*-statistic	99.1574	48.2939	41.8126	30.5544	48.2939
Prob (F-statistic)	0.0000	0.0000	0.0000	0.0000	0.0000
**Panel B: Determinants of investment abroad concentration (*IAC*):**					
	**(6)**	**(7)**	**(8)**	**(9)**	**(10)**
	***IAC***	***IAC***	***AL_IAC***	**ex. USA**	**ex. CHN**
*UAI*		0.0030 (9.0036)***	0.0209 (9.0485)***	0.0009 (1.9026)*	0.0030 (9.0036)***
*perGDP*	-3.73E-09 (-4.0028)***	-2.09E-09 (-2.5448)**	-2.00E-08 (-4.5536)***	-4.11E-09 (-4.3639)***	-2.09E-09 (-2.5448)**
*GDP growth*	-0.0043 (-2.0399)**	-0.0059 (-3.0750)***	-0.0342 (-2.9108)***	-0.0040 (-1.8534)*	-0.0059 (-3.0750)***
*ln (ST / GDP)*	-0.0512 (-6.5142)***	-0.0398 (-5.4204)***	-0.2883 (-5.9985)***	-0.0489 (-5.7869)***	-0.0398 (-5.4204)***
*ln (MC / GDP)*	0.0828 (7.8743)***	0.1072 (8.9542)***	0.7358 (8.8099)***	0.1019 (8.4904)***	0.1072 (8.9542)***
*ln (FC / GDP)*	-0.1082 (-5.7905)***	-0.1224 (-8.4994)***	-1.0781 (-12.095)***	-0.1589 (-9.6594)***	-0.1224 (-8.4994)***
*MC*	-4.04E-15 (-6.4426)***	-1.84E-15 (-3.5396)***	-2.84E-14 (-7.2355)***	3.62E-14 (5.5812)***	-1.84E-15 (-3.5396)***
*constant*	-0.1061 (-1.8964)***	-0.3946 (-7.9827)***	-1.1601 (-3.8861)***	-0.0572 (-0.7128)***	-0.3946 (-7.9827)***
Adjusted *R*-squared	0.7562	0.8387	0.9059	0.6196	0.8387
S.E. of regression	0.1105	0.1039	0.6872	0.1079	0.1039
*F*-statistic	101.2884	145.1243	267.6616	41.9531	145.1243
Prob (*F*-statistic)	0.0000	0.0000	0.0000	0.0000	0.0000

Note: This table denotes the estimation results of uncertainty avoidance impacts on the home bias (*HB*) and investment abroad concentration (*IAC*) according to Eq (4). Yearly data are employed for the period 2001 to 2018. GLS with cross-section weights and the PCSE method are employed to perform the results. Explanatory and control variables include the *UAI* (culture of uncertainty avoidance), *perGDP* (GPD per capita), *GDP growth* (GDP growth rate), *ln (ST / GDP)* (the logarithm of stock traded to GDP), *ln (MC / GDP)* (the logarithm of the market capitalization of listed companies to GDP), *ln (FC / GDP)* (the logarithm of domestic credit provided by the financial sector to GDP), *MC* (market capitalization of listed companies).

*, **, *** indicate significance at the 10%, 5% and 1% levels, respectively.

#### 4.1.2 Discussions

Hypotheses 1 and 2, stating that uncertainty avoidance is positively correlated to the degree of underdiversification are confirmed. When uncertainty avoidance increases by one standard deviation (23.25), based on Models 2 and 7, the degrees of home bias and the concentration of investments abroad increase on average by 6.42 and 0.07 units, respectively. Models 3 and 8 with alternative variables confirm these findings. Furthermore, the results on the home bias are in line with [19, 20], although the effects of uncertainty avoidance on the home bias are greater than those presented in [20]. This may be because these two studies focused only on institutional investors’ bias, whereas this paper also considers individual investors who are more susceptible to irrational factors and therefore exhibit a greater behavioral bias than institutional investors.

Uncertainty avoidance is a significant explanatory factor for investment with underdiversification. The significance of some economic and financial factors decreases when a cultural variable is introduced into the model. Compared to Model 1, the significance of economic growth and market capitalization of listed companies, acting as variables explaining investor’s home bias in Model 2, fell to the 10% and 5% levels, respectively. Similarly, compared to Model 6, the significance of GDP per capita, acting as the variable explaining investors’ concentration of investments abroad in Model 7, fell to the 10% level. This effect of cultural variables on the explanatory power of economic variables has been noted in extant research. [11] examined culture and stock price synchronization, and found that the effects of economic development on stock price synchronization are almost non-existent when cultural variables are introduced into models. A possible interpretation for the high explanatory power of cultural variables offers institutional theory, stating that culture, as the basis of normative and cognitive power, puts constraints on the behavior of members of society.

The world’s largest investment market, the United States, was removed from the sample to obtain Models 4 and 9, and the world’s fastest-growing investment market, China, was removed from the sample to obtain Models 5 and 10, in order to avoid the excessive influence of a single economy. A comparison of Models 2, 4, 7, and 9 reveals that the US market distorts the overall results, as the effect of US investors’ ambiguity aversion (proxied by uncertainty avoidance) on the home bias is below average, and the effect on the concentration of investments abroad is above average. Models 2, 5, 7, and 10 show that the Chinese market does not affect the overall results, as the coefficients of uncertainty avoidance remain the same regardless of whether or not the sample includes China. Compared with Chinese investors, uncertainty avoidance of US investors show more diversities, which is contrary to our stereotype but has been confirmed in the literature [58], found that the Chinese exhibited significantly less probabilistic thinking and more ambiguous gambling decisions, so they are more like to perform risk-taking behaviors.

In line with the prediction, the high level of economic and financial development of home countries provides the investor with more accurate information and more reliable channels for investing abroad, and thus decreases the concentration of investment abroad. Contrary to results in [20], the level of economic development is positively correlated to home bias. The reason for this difference in conclusions is that [20] focused only on institutional investors, whereas the sample used in this paper includes both institutional and individual investors, and the economic boom can get many people interested in investing in the domestic market and become individual investors. The effects of financial development on the home bias are basically consistent with the prediction, as the ratio of market capitalization of listed companies to GDP, ratio of domestic credit provided by financial sector to GDP and market capitalization of listed companies are all negatively correlated to home bias. The high ratio of stock traded to GDP is positively increases home bias, since a liquid market is considered by investors to be healthier and safer.

### 4.2 Robustness tests

#### 4.2.1 Alternating measure of uncertainty avoidance

Although Hofstede’s national culture dimensions are widely accepted in the literature, it is important to ensure that the findings are not sensitive to the choice of uncertainty avoidance. Therefore, *UAI* in Eq (4) (*UAI*-*UNDV* model) is replaced by three alternative variables, *TK_UAI*, *GLOBE_UAI*, and *AA_UAI*, and the regression is performed. Due to lack of data, 8 countries had to be removed from the sample for the regression with the alternative variable *TK_UAI*, and 13 countries could not be included in the sample for the regression with the alternative variable *GLOBE_UAI*. The regression results are reported in Table 5.

**Table 5:** The robustness results from the alternating measure of uncertainty avoidance.

**Panel A: alternating *UAI* with *TK_ UAI***				
	**(11)**	**(12)**	**(13)**	**(14)**
	***HB***	***AL_HB***	***IAC***	***AL_IAC***
*TK_UAI*	0.1699 (3.4262)***	0.1694 (3.4545)***	0.0024 (6.1675)***	0.0155 (5.9168)***
*perGDP*	1.71E-07 (5.7964)***	1.72E-07 (5.8672)***	-4.17E-09 (-4.7316)***	-3.52E-08 (-7.6361)***
*GDP growth*	0.3987 (2.0418)**	0.3921 (2.0244)**	-0.0052 (-2.6878)***	-0.0288 (-2.3901)**
*ln (ST / GDP)*	3.4786 (4.9528)***	3.5605 (5.1167)***	-0.0415 (-5.1978)***	-0.2842 (-5.6567)***
*ln (MC / GDP)*	0.6315 (0.4881)	0.4468 (0.3493)	0.0984 (8.1358)***	0.6525 (8.0017)***
*ln (FC / GDP)*	-9.2406 (-7.2081)***	-9.0901 (-7.1396)***	-0.0980 (-6.8769)***	-0.9232 (-10.6973)***
*MC*	-3.95E-13 (-3.6484)***	-2.50E-13 (-2.3828)***	-2.36E-15 (-4.2777)***	-3.43E-14 (-7.8336)***
*constant*	103.4309 (14.9774)***	103.3636 (15.1532)***	-0.4074 (-6.6689)***	-1.0608 (-2.7727)***
Adjusted *R*-squared	0.6510	0.6143	0.8246	0.8928
S.E. of regression	15.1393	15.0201	0.1073	0.7073
*F*-statistic	52.1590	44.6797	129.9897	229.4703
Prob (*F*-statistic)	0.0000	0.0000	0.0000	0.0000
**Panel B: alternating *UAI* with *GLOBE_UAI***				
	**(15)**	**(16)**	**(17)**	**(18)**
	***HB***	***AL_HB***	***IAC***	***AL_IAC***
*GLOBE_UAI*	11.7005 (7.5493)***	11.6251 (-7.5663)***	0.0687 (6.5923)***	0.8317 (14.8356)***
*perGDP*	6.39E-08 (3.1239)***	6.48E-08 (3.1999)***	-2.80E-09 (-2.9005)***	-2.62E-08 (-5.1782)***
*GDP growth*	0.1709 (2.0677)**	0.1687 (2.0648)**	-0.0036 (-1.5649)	-0.0309 (-2.2840)**
*ln (ST / GDP)*	1.4307 (3.5632)***	1.4233 (3.5819)***	-0.0386 (-3.7014)***	-0.2782 (-4.0025)***
*ln (MC / GDP)*	2.2033 (2.6932)***	2.1247 (2.6299)***	0.0483 (4.4082)***	0.1825 (2.5701)**
*ln (FC / GDP)*	-7.8033 (-8.7476)***	-7.6945 (-8.7387)***	-0.0636 (-3.7965)***	-0.6732 (-6.4157)***
*MC*	-7.93E-13 (-13.5334)***	-6.34E-13 (-11.694)***	-6.28E-15 (-7.3221)***	-5.49E-14 (-9.0994)***
*constant*	162.3493 (29.714)***	161.8745 (29.9640)***	-0.4817 (-9.2853)***	-2.5941 (-8.0811)***
Adjusted *R*-squared	0.8714	0.8609	0.7164	0.8485
S.E. of regression	6.0341	5.9553	0.0930	0.6016
*F*-statistic	132.6543	121.2042	50.4420	110.5718
Prob (*F*-statistic)	0.0000	0.0000	0.0000	0.0000
**Panel C: alternating *UAI* with *AA_UAI***				
	**(19)**	**(20)**	**(21)**	**(22)**
	***HB***	***AL_HB***	***IAC***	***AL_IAC***
*AA_UAI*	11.0505 (7.0065)***	35.0208 (7.0016)***	1.0711 (1.7741)*	0.1202 (1.4297)
*perGDP*	1.45E-07 (2.7872)***	3.99E-07 (2.5744)**	2.98E-08 (3.3740)***	3.70E-09 (2.9092)***
*GDP growth*	0.1108 (2.0825)**	0.2773 (1.6592)*	-0.0085 (-0.4815)	-0.0008 (-0.3086)
*ln (ST / GDP)*	0.0223 (7.4974)***	0.0615 (4.6595)***	-0.0049 (-5.3797)***	-0.0005 (-4.9025)***
*ln (MC / GDP)*	-0.0219 (-4.8814)***	-0.0669 (-4.5965)***	-0.0067 (-4.9477)***	-0.0006 (-3.5732)**
*ln (FC / GDP)*	-0.0115 (-8.4252)***	-7.6945 (-7.0409)***	-0.0046 (-6.7863)***	-0.0004 (-4.8356)***
*MC*	-6.04E-13 (-8.2901)***	-9.75E-12 (-6.3162)***	-8.24E-15 (-5.1190)***	-3.18E-14 (-4.1573)***
*constant*	-3.8559 (-5.9568)***	-12.3580 (-5.6885)***	-2.0939 (-5.2965)***	-0.3877 (-6.9748)***
Adjusted *R*-squared	0.3337	0.3113	0.8875	0.8341
S.E. of regression	5.5033	18.2634	0.8019	0.1174
*F*-statistic	13.5209	12.3051	198.3703	120.7271
Prob (*F*-statistic)	0.0000	0.0000	0.0000	0.0000

Note: This table denotes the robustness test using the alternating measure. Yearly data are employed for the period 2001 to 2018. GLS with cross-section weights and the PCSE method are employed to perform the results. Explanatory and control variables include the *TK_UAI* (alternative culture of uncertainty avoidance calculated by Tang and Koveos (2008)), *GLOBE_UAI* (alternative culture of uncertainty avoidance calculated by the project of House et al. (2004)), *AA_UAI* (alternative culture of uncertainty avoidance calculated by Rieger et al. (2015)), *perGDP* (GPD per capita), *GDP growth* (GDP growth rate), *ln (ST / GDP)* (the logarithm of stock traded to GDP), *ln (MC / GDP)* (the logarithm of the market capitalization of listed companies to GDP), *ln (FC / GDP)* (the logarithm of domestic credit provided by financial sector to GDP), *MC* (market capitalization of listed companies). *, **, *** indicate significance at the 10%, 5% and 1% levels, respectively.

Models with the alternating measure of uncertainty avoidance confirm the previous conclusion that uncertainty avoidance is positively correlated to the degree of investment underdiversification. When uncertainty avoidance proxied by *TK_UAI* increases by one standard deviation (23.42), then, based on Models 11 and 13, the degrees of home bias and concentration of investments increase on average by 3.98 and 0.06 units, respectively, and Models 12 and 14 with alternative variables confirm these findings. When uncertainty avoidance proxied by *GLOBE_UAI* increases by one standard deviation (0.66), then, based on Models 15 and 17, the degrees of home bias and concentration of investments increase on average by 7.79 and 0.05 units, respectively, and Models 16 and 18 with alternative variables confirm these findings. Besides, when uncertainty avoidance proxied by *AA_UAI* increases by one standard deviation (0.09), then, based on Models 19 and 21, the degrees of home bias and concentration of investments increase on average by 0.99 and 0.10 units, respectively, and Models 20 and 22 with alternative variables confirm these findings.

#### 4.2.2 Endogeneity

Although this paper selects a set of control variables representing the macroeconomic and macro-financial development, the *UAI-UNDV* model may still have endogenous problems caused by omitted variables, such as investor’s overconfidence, reference point, and experience. Therefore, the robustness tests using the Instrumental Variable (IV) approach are performed to address these endogeneity concerns. The goal of the instrumental variable method is to select a variable that is correlated with the endogenous independent variable but does not directly affect the dependent variable, so that the instrumental variable satisfies the exogeneity and relevance conditions [59].

*Religiosity* is selected as the first IV for uncertainty avoidance. [14] argued that religion helps people cope with the unpredictable future by giving them ultimate certainty, and it alleviates members’ uncertainty about the future by providing them with future guidance. Religious faith, same as culture, is also an important feature that distinguishes groups, as individuals are likely to identify with the religion of society due to their connection with that society [60, 61]. The data on *religiosity* were obtained from [61], which considered religiosity from three dimensions: the member of the religion, religious importance, and religious services.

The second IV for uncertainty avoidance used in this paper is *2*^*nd*^ *person-pronoun*, defined as the proportion of a country’s population that speaks languages with more than one second-person singular pronoun. [62] found that compared to people who speak languages with only one second-person singular pronoun, those who speak languages with two or more second-person singular pronouns have a higher degree of uncertainty avoidance, as they face higher pressure from social interactions to select the appropriate second-person singular pronoun. The data on *2*^*nd*^ *person-pronoun* were obtained from [63]. Results of the Two-Stage Least Square (2SLS) regression with IVs are reported in Table 6.

**Table 6:** The robustness results using the 2SLS method with IVs.

**Panel A: 2SLS using *religiosity* as IV**						
	**(23)**		**(24)**	**(25)**	**(26)**	**(27)**
	***UAI***		***HB***	***AL_HB***	***IAC***	***AL_IAC***
**First-stage (OLS)**		**Second-stage**				
*religiosity*	-0.8008 (-13.7027)***	*predicted UAI*	0.9803 (6.4023)***	0.9681 (6.4065)***	0.0151 (10.6852)***	0.0915 (7.8135)***
*constant*	5.1598 (84.2370)***	*perGDP*	7.67E-07 (5.6517)***	7.60E-07 (5.6869)***	4.88E-09 (3.1131)***	2.63E-08 (2.2865)**
Adjusted *R*-squared	0.0332	*GDP growth*	0.1513 (0.7442)	0.1427 (0.7103)	-0.0070 (-2.2842)**	-0.0448 (-2.6010)**
S.E. of regression	19.2721	*ln (ST / GDP)*	7.8305 (9.1050)***	7.8465 (9.2491)***	-0.0935 (-6.9095)***	-0.5026 (-6.4609)***
*F*-statistic	18.2695	*ln (MC / GDP)*	9.6783 (4.2054)***	9.4632 (4.1547)***	0.2101 (6.8535)***	1.2813 (7.6275)***
Prob(*F*-statistic)	0.0000	*ln (FC / GDP)*	-19.3973 (-8.0517)***	-19.2799 (-8.1300)***	-0.0195 (-0.4995)	-0.4885 (-2.2246)**
		*MC*	2.40E-13 (1.2243)	3.84E-13 (1.9776)**	8.10E-15 (4.7441)***	2.54E-14 (2.5323)**
		*constant*	37.6459 (1.9025)*	38.865 (2.0005)**	-1.9471 (-8.2452)***	-10.3259 (-5.9500)***
		Prob (*F*-statistic)	0.0000	0.0000	0.0000	0.0000
**Panel B: 2SLS using *2***^***nd***^ ***person-pronoun* as IV**						
	**(28)**		**(29)**	**(30)**	**(31)**	**(32)**
	***UAI***		***HB***	***AL_HB***	***IAC***	***AL_IAC***
**First-stage (OLS)**		**Second-stage**				
*2*^*nd*^ *person-pronoun*	0.4433 (6.7876)***	*predicted UAI*	0.6414 (15.0800)***	0.6408 (15.2789)***	0.0050 (13.9988)***	0.0310 (14.8884)***
*constant*	4.1757 (141.3723)***	*perGDP*	5.21E-07 (9.1709)***	5.22E-07 (9.2303)***	-1.91E-09 (-2.2944)***	-1.59E-08 (-3.6256)***
Adjusted *R*-squared	0.0669	*GDP growth*	0.2009 (1.2937)	0.1923 (1.2495)	-0.0059 (-3.0436)***	-0.0342 (-3.1632)***
S.E. of regression	0.6554	*ln (ST / GDP)*	5.3190 (9.1717)***	5.3665 (9.3753)***	-0.0229 (-3.2058)***	-0.2374 (-5.0450)***
*F*-statistic	46.0712	*ln (MC / GDP)*	2.2881 (1.9086)*	2.2020 (1.8536)*	0.1279 (9.9466)***	0.8565 (10.3752)***
Prob(*F*-statistic)	0.0000	*ln (FC / GDP)*	-15.2387 (-13.3660)***	-15.1680 (-13.422)***	-0.1580 (-12.1735)***	-1.2351 (-15.8631)***
		*MC*	2.42E-13 (1.7973)*	3.92E-13 (2.9468)***	-6.85E-16 (-1.2210)***	-1.93E-14 (-5.3448)***
		*constant*	80.0506 (11.5935)***	80.0414 (11.7575)***	-0.5118 (-10.3920)***	-1.8409 (-6.9050)***
		Prob (*F*-statistic)	0.0000	0.0000	0.0000	0.0000

Note: This table denotes the endogeneity robustness test. Yearly data are employed for the period 2001 to 2018. The regression method of 2SLS with IVs is employed to perform the results. Two instrumental variables adopted are *religiosity* and *2*^*nd*^ *person-pronoun*. Control variables include the *perGDP* (GPD per capita), *GDP growth* (GDP growth rate), *ln (ST / GDP)* (the logarithm of stock traded to GDP), *ln (MC / GDP)* (the logarithm of the market capitalization of listed companies to GDP), *ln (FC / GDP)* (the logarithm of domestic credit provided by the financial sector to GDP), *MC* (market capitalization of listed companies).

*, **, *** indicate significance at the 10%, 5% and 1% levels, respectively.

The Ordinary Least Squares (OLS) method is adopted for the first-stage regression, where *UAI* is regressed on instrumental variables, *religiosity*, and *2*^*nd*^ *person-pronoun*, in order to obtain the *predicted UAI*. As the coefficients of instrumental variables are both significant at the 1% level, the choice of instrumental variables is appropriate. In the second stage of regression, *predicted UAI* is introduced into Eq (4) (the *UAI*-*UNDV* model) as explanatory variables for further regression analysis. Since the results of the second stage regression reported in Table 6 are almost identical to those reported in Table 4, the results of the *UAI*-*UNDV* model are not affected by the problem of endogeneity.

### 4.3 Internal mechanism of uncertainty avoidance and investment underdiversification

To test Hypothesis 3, this paper constructs the Ambiguity-Underdiversification (*AB-UNDV*) model that can be expressed in Eq (5).

$$\begin{array}{l} UNDV_{i,t}=\beta_{0}+\beta_{1}AB_{i,t}+\beta_{2}perGDP_{i,t}+\beta_{3}GDP\;growth_{i,t}+\beta_{4}\ln{\left(ST/GDP\right)}_{i,t}\\ \;+\beta_{5}\ln{\left(MC/GDP\right)}_{i,t}+\beta_{6}\ln{\left(FC/GDP\right)}_{i,t}+\beta_{7}MC_{i,t}+u_{i,j,t}, \end{array}$$ (5)

where *AB*_*i*,*t*_ denotes the ambiguity of country *i* in year *t*, proxied by the political stability of country *i* in year *t PS*_*i*,*t*_, economic freedom of country *i* in year *t EF*_*i*,*t*_ and the rule of law of country *i* in year *t RL*_*i*,*t*_.

To test Hypothesis 4, this paper constructs the Uncertainty Avoidance-Ambiguity-Underdiversification (*AB-UAI-UNDV*) model that can be expressed by Eq (6).


$$\begin{array}{l} UNDV_{i,t}=\beta_{0}+\beta_{1}AB_{i,t}+\beta_{2}UAI_{i}+\beta_{3}AB_{i,t}\times UAI_{i}+\beta_{4}perGDP_{i,t}+\beta_{5}GDP\;growth_{i,t}+\beta_{6}\ln{\left(ST/GDP\right)}_{i,t}\\ \;+\beta_{7}\ln{\left(MC/GDP\right)}_{i,t}+\beta_{8}\ln{\left(FC/GDP\right)}_{i,t}+\beta_{9}MC_{i,t}+u_{i,j,t}, \end{array}$$ (6)

The moderating effect of uncertainty avoidance can be better described by Eq (7), a modified version of Eq (6).


$$\begin{array}{l} UNDV_{i,t}=\beta_{0}+(\beta_{1}+\beta_{3}UAI_{i})\times AB_{i,t}+\beta_{2}UAI_{i}+\beta_{4}perGDP_{i,t}+\beta_{5}GDP\;growth_{i,t}+\beta_{6}\ln{\left(ST/GDP\right)}_{i,t}\\ \;+\beta_{7}\ln{\left(MC/GDP\right)}_{i,t}+\beta_{8}\ln{\left(FC/GDP\right)}_{i,t}+\beta_{9}MC_{i,t}+u_{i,j,t}, \end{array}$$ (7)

As can be seen from Eq (7), the overall effect of ambiguity *AB*_*i*,*t*_ on investment underdiversification *UNDV*_*i*,*t*_ can be influenced by uncertainty avoidance *UAI*_*i*_, *β*_*1*_+*β*_*3*_*UAI*_*i*_. Based on Hypotheses 3 and 4, this paper predicts *β*_*1*_<0 and *β*_*3*_>0. The regression results are reported in Table 7.

**Table 7:** The results of *AB-UNDV* and *AB-UAI-UNDV* models.

**Panel A: The effects of ambiguity (*AB*) and uncertainty avoidance (*UAI*) on the home bias (*HB*)**						
	***political stability* (*PS*)**		***economic freedom* (*EF*)**		***rule of law* (*RL*)**	
	**(33)**	**(34)**	**(35)**	**(36)**	**(37)**	**(38)**
*IA*	-11.4530 (-14.0567)***	-34.0369 (-13.2776)***	-0.6139 (-4.9026)***	-1.1761 (-3.2471)***	-17.3292 (-14.6976)***	-27.6251 (-12.1691)***
*UAI*		0.0388 (1.0781)		-0.1237 (-0.3482)		0.0346 (1.2230)
*IA×UAI*		0.3421 (10.4999)***		0.0058 (1.1002)		0.1713 (5.3680)***
*perGDP*	2.29E-07 (5.8390)***	1.79E-08 (0.3608)	7.76E-08 (1.6497)	1.54E-07 (2.5762)**	1.17E-07 (2.8243)***	6.39E-08 (1.4067)
*GDP growth*	0.3941 (2.4773)**	0.3532 (2.2027)**	0.7509 (3.4024)***	0.5749 (2.5909)**	0.8864 (5.3853)***	0.6584 (4.8356)***
*ln (ST / GDP)*	0.1119 (0.1659)	2.1937 (3.6640)***	3.1934 (4.4158)***	2.8602 (3.8414)***	2.1329 (3.4395)***	2.5975 (4.6989)***
*ln (MC / GDP)*	0.3266 (0.3845)	-2.0495 (-2.0192)**	-1.9948 (-2.0144)**	2.3840 (2.0390)**	-4.4145 (-5.1732)***	-4.4545 (-5.5417)***
*ln (FC / GDP)*	0.2056 (0.1100)	-3.8547 (-2.6059)***	-5.8446 (-3.5911)***	-8.8792 (-5.9172)***	6.3018 (3.6030)***	3.7363 (2.4480)**
*MC*	-2.92E-13 (-4.1362)***	-1.76E-13 (-2.4347)***	-3.48E-13 (-3.2490)***	5.41E-14 (0.3590)	1.88E-13 (1.6398)	3.70E-13 (3.0585)***
*constant*	80.0369 (13.0605)***	100.9318 (16.8827)***	151.2034 (17.8756)***	166.8880 (6.5376)***	70.6336 (11.3363)***	79.5892 (12.6228)***
Adjusted *R*-squared	0.7382	0.7465	0.5494	0.6614	0.7942	0.8015
Prob (*F*-statistic)	0.0000	0.0000	0.0000	0.0000	0.0000	0.0000
**Panel B: The effects of ambiguity (*AB*) and uncertainty avoidance (*UAI*) on investment abroad concentration (*IAC*)**						
	***political stability* (*PS*)**		***economic freedom* (*EF*)**		***rule of law* (*RL*)**	
	**(39)**	**(40)**	**(41)**	**(42)**	**(43)**	**(44)**
*IA*	-0.0143 (-1.6739)*	-0.0565 (-1.8797)	-0.0031 (-3.8649)***	0.0177 (1.5605)	-0.0489 (-5.6350)***	0.0444 (1.2624)
*UAI*		0.0033 (8.5594)***		0.0261 (6.8111)***		0.0040 (6.1074)***
*IA×UAI*		-0.0009 (-2.2238)*		-0.0003 (-5.9407)***		-0.0014 (-2.6716)***
*perGDP*	-3.74E-09 (-4.1240)***	-1.56E-09 (-1.9471)**	-3.40E-09 (-3.4271)***	-1.66E-10 (-0.2014)	-3.16E-09 (-3.4196)***	-1.04E-09 (-1.1664)
*GDP growth*	-0.0048 (-2.5473)**	-0.0062 (-3.1189)**	-0.0044 (-2.3498)**	-0.0065 (-2.9563)**	-0.0040 (-2.3333)**	-0.0049 (-2.5367)**
*ln (ST / GDP)*	-0.0569 (-7.4698)***	-0.0418 (-5.5116)***	-0.0530 (-7.0728)***	-0.0336 (-4.6040)***	-0.0571 (-8.1152)***	-0.0398 (-5.6928)***
*ln (MC / GDP)*	0.0881 (8.2337)***	0.1113 (8.9391)***	0.0763 (7.5630)***	0.1418 (11.4617)***	0.0731 (6.8598)***	0.1085 (8.8790)***
*ln (FC / GDP)*	-0.0976 (-4.5303)***	-0.1095 (-6.0178)***	-0.0849 (-4.5083)***	-0.1116 (-7.7187)***	-0.0533 (-2.4697)**	-0.0565 (-3.0345)***
*MC*	-3.57E-15 (-5.7472)***	-2.00E-15 (-3.7155)***	-3.02E-15 (-5.9470)***	-3.77E-15 (-3.9166)***	-2.30E-15 (-4.4016)***	-2.30E-15 (-3.9002)***
*constant*	-0.1611 (-2.3830)**	-0.4887 (-6.8176)***	0.0291 (0.4234)	-1.8822 (-6.2949)***	-0.2858 (-4.5926)***	-0.7600 (-6.9937)***
Adjusted *R*-squared	0.7856	0.8660	0.8039	0.7592	0.8252	0.8630
Prob (*F*-statistic)	0.0000	0.0000	0.0000	0.0000	0.0000	0.0000

Note: This table denotes the estimation results of uncertainty avoidance moderating impacts on the home bias (*HB*) and investment abroad concentration (*IAC*) according to Eqs (5), (6), and (7). Yearly data are employed for the period 2001 to 2018. GLS with cross-section weights and the PCSE method are employed to perform the results. Ambiguity (*AB*) is presented from three aspects, political stability (*PS*), economic freedom (*EF*), and the rule of law (*RL*). Explanatory and control variables include the *UAI* (culture of uncertainty avoidance), *perGDP* (GPD per capita), *GDP growth* (GDP growth rate), *ln (ST / GDP)* (the logarithm of stock traded to GDP), *ln (MC / GDP)* (the logarithm of the market capitalization of listed companies to GDP), *ln (FC / GDP)* (the logarithm of domestic credit provided by the financial sector to GDP), *MC* (market capitalization of listed companies).

*, **, *** indicate significance at the 10%, 5% and 1% levels, respectively.

Hypothesis 3, stating that cross-border ambiguity is positively correlated to the degree of investment underdiversification, is confirmed. When the index of political stability (*PS*) increases by one standard deviation (ambiguity decrease), then, based on Models 33 and 39, the degrees of home bias and the concentration of investments abroad decreases on average by 10.80 and 0.01 units, respectively. Similarly, when indexes of economic freedom (*EF*) and the rule of law (*RL*) increase by one standard deviation (ambiguity decrease), then, based on Models 35 and 37, the degree of home bias decreases on average by 5.82 and 16.61 units, respectively, and based on Models 41 and 43, the degree of concentration of investment abroad decreases on average by 0.03 and 0.05 units respectively. The effects of ambiguity on the home bias are clearly stronger than those on the concentration of investments abroad.

Hypothesis 4, stating that uncertain avoidance moderates the effect of ambiguity on investment underdiversification, is confirmed. When uncertainty avoidance increases by one standard deviation, then, based on Models 34, 36, and 38, the degree of effect of ambiguity on home bias increases on average by 7.95, 0.13, and 3.99 units, respectively, and based on Models 40, 42 and 44, the degree of effect of ambiguity on the concentration of investment abroad concentration increases on average by 0.02, 0.01, and 0.03 units respectively.

**Fig 1:**
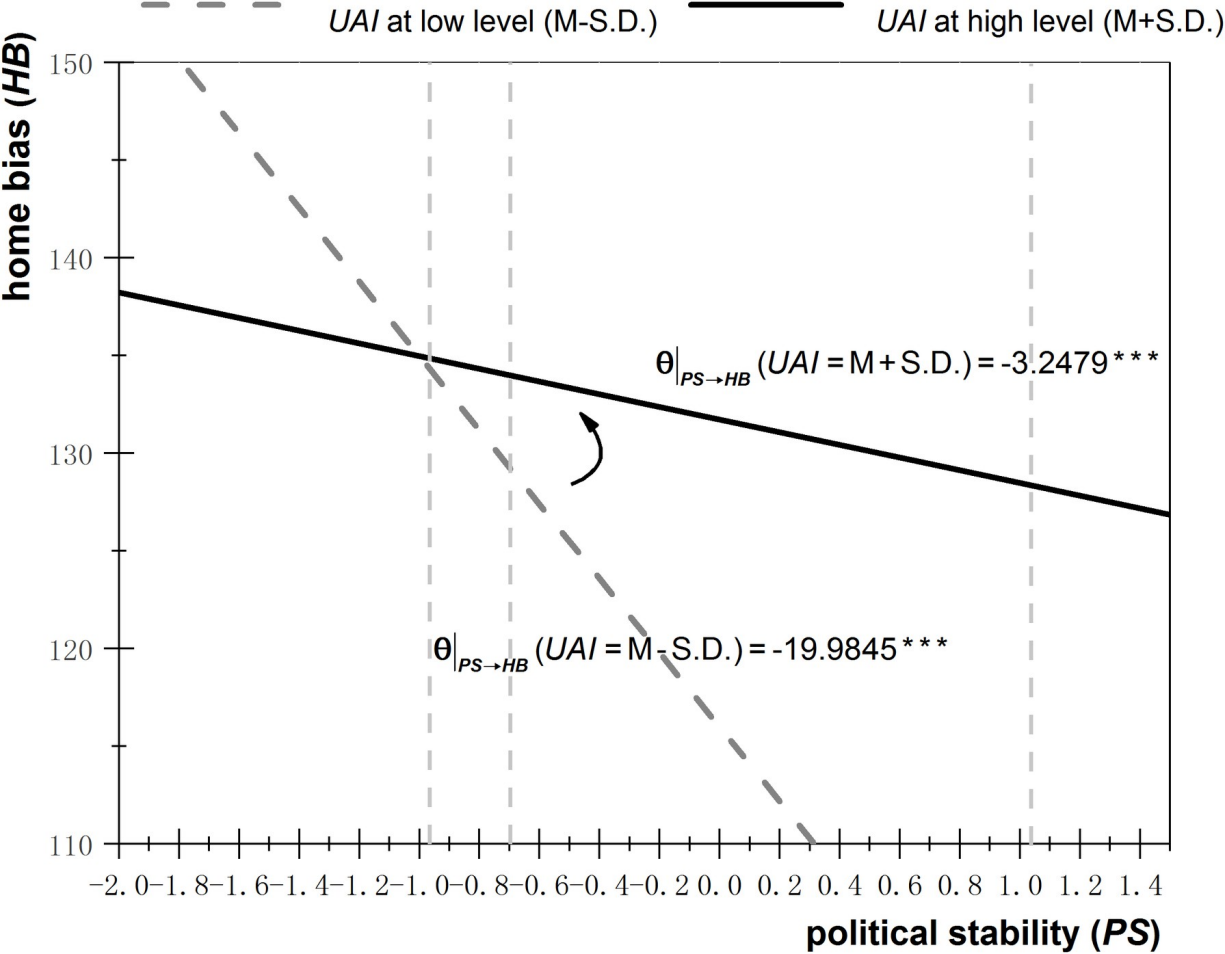
Effects of political stability on home bias under different degrees of uncertainty avoidance.

**Fig 2:**
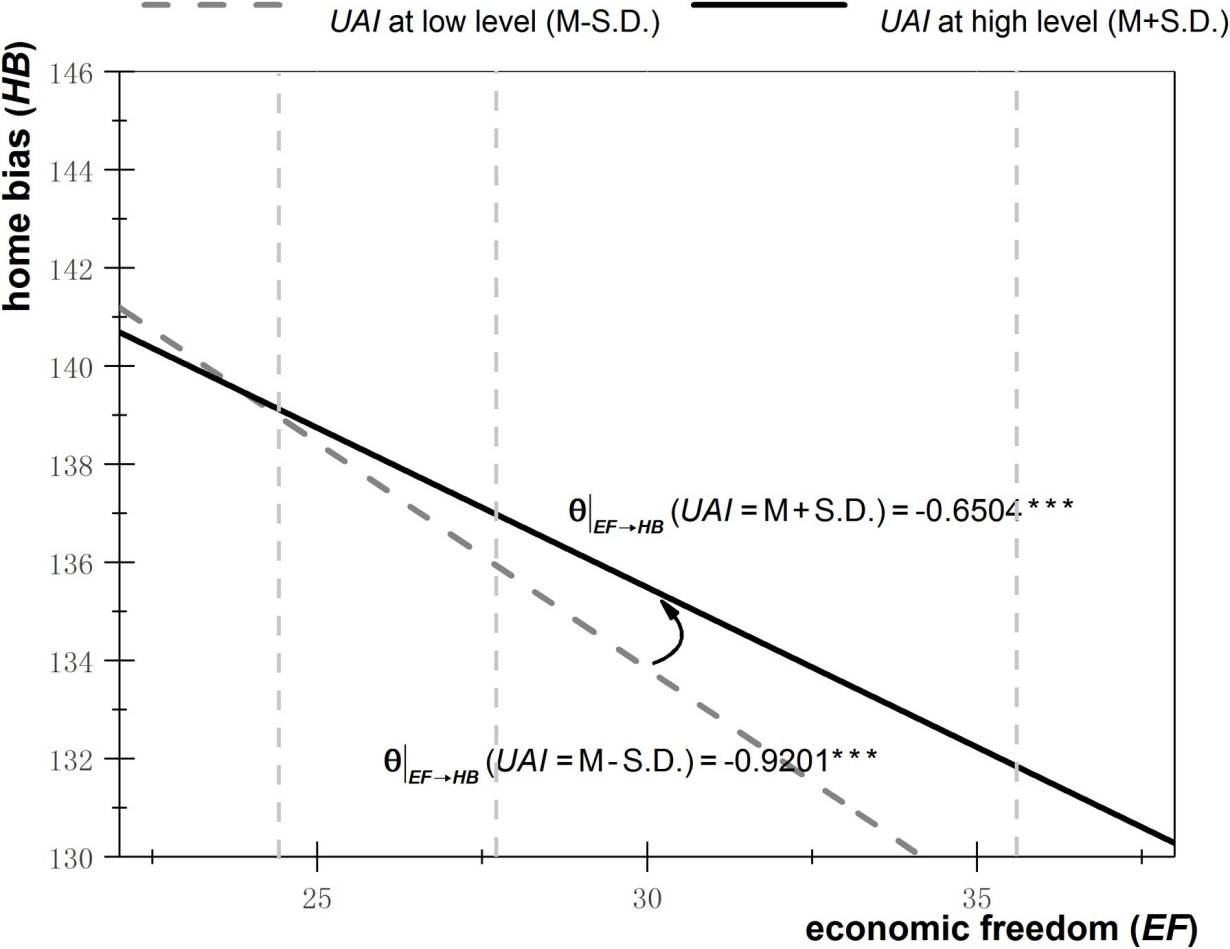
Effects of economic freedom on home bias under different degrees of uncertainty avoidance.

**Fig 3:**
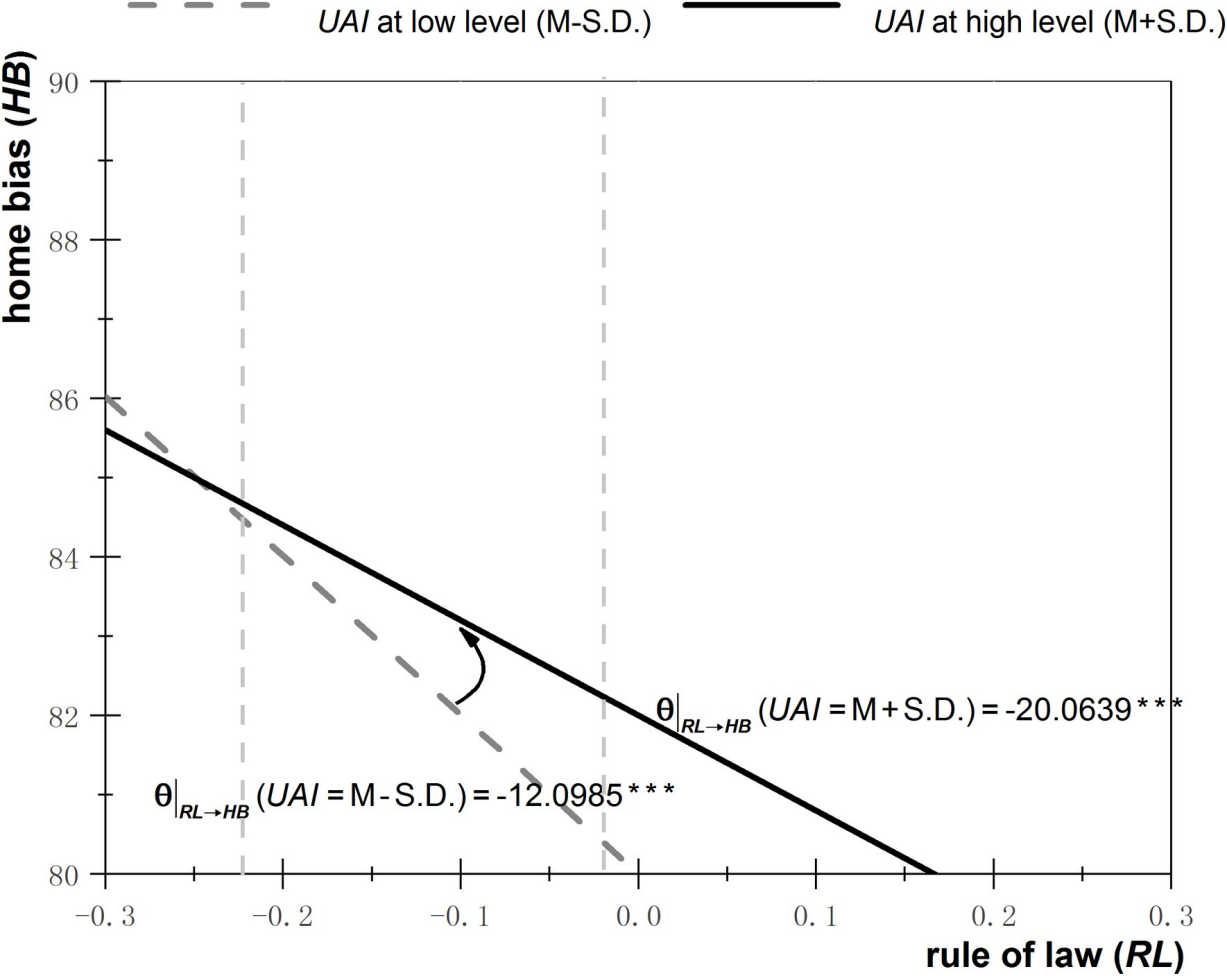
Effects of the rule of law on home bias under different degrees of uncertainty avoidance.

Figs 1–3 depict the effects of ambiguity (including *PS*, *EF*, and *RL*) on home bias under different degrees of uncertainty avoidance. The high degree of uncertainty avoidance is set as the mean value plus one standard deviation, while the low degree is set as the mean value minus the standard deviation. These values are substituted into Eq, (7), and regressions are performed. The solid line represents the effects of ambiguity on home bias under a high degree of uncertainty avoidance, whereas the effects of ambiguity on home bias under a low degree of uncertainty avoidance are depicted by the dotted line.

As can be seen from Fig 1, when uncertainty avoidance is relatively high (solid line), then a decrease of ambiguity (an increase of *PS*) by one unit leads to a reduction of the degree of home bias by 3.25 units, whereas when uncertainty avoidance is relatively low (dotted line), then a decrease of ambiguity (an increase of *PS*) by one unit causes a reduction of the degree of home bias by 19.98 units. As the degree of uncertainty avoidance shifts from low to high (from dotted to solid line), the effect of ambiguity reduction on home bias decline diminishes, indicating that uncertainty avoidance positively moderates the relationship between ambiguity and investment underdiversification. A similar moderating effect of uncertainty avoidance can be seen in Figs 2 and 3.

To test Hypothesis 5, this paper constructs the Uncertainty Avoidance-Status Quo Bias (*UAI-SQB*) model and the Uncertainty Avoidance-Status Quo Bias-Underdiversification (*UAI-SQB-UNDV*) model that can be expressed by Eq (8) and Eq (9), respectively.

$$SQB_{i}=\beta_{0}+\beta_{1}UAI_{i}+\beta_{2}perGDP_{i,t}+u_{i,j,t},$$ (8)

$$\begin{array}{l} UNDV_{i,t}=\beta_{0}+\beta_{1}SQB+\beta_{2}UAI_{i}+\beta_{3}perGDP_{i,t}+\beta_{4}GDP\;growth_{i,t}+\beta_{5}\ln{\left(ST/GDP\right)}_{i,t}\\ \;+\beta_{6}\ln{\left(MC/GDP\right)}_{i,t}+\beta_{7}\ln{\left(FC/GDP\right)}_{i,t}+\beta_{8}MC_{i,t}+u_{i,j,t}, \end{array}$$ (9)

where *SQB*_*i*_ denotes the degree of status quo bias of country *i*, including *HB_SQB*_*i*_ and *IAC_SQB*_*i*_. The regression results are reported in Table 8.

**Table 8:** The results of *UAI-SQB* and *UAI-SQB-UNDV* models.

**Panel A: The effects of uncertainty avoidance (*UAI*) and status quo bias (*SQB*) on the home bias (*HB*)**				
	***UAI-HB_SQB***			***UAI-HB_SQB*-*HB***
	**(45)**		**(46) *HB***	**(47) *AL_HB***
*UAI*	0.0004 (19.603)***	*HB_SQB*	37.443 (15.7442)***	37.3571 (15.8754)***
*perGDP*	5.38E-09 (20.832)***	*UAI*	0.1949 (4.1653)***	0.1917 (4.1591)***
*constant*	0.6711 (10.6342)***	*perGDP*	7.02E-07 (12.6559)***	7.01E-07 (12.8048)***
*R*-squared	0.1780	*GDP growth*	0.5173 (3.3230)***	0.5111 (3.3299)***
Adjusted *R*-squared	0.1757	*ln (ST / GDP)*	-3.8565 (-6.4420)***	-3.8253 (-6.4003)***
SE of regression	0.3308	*ln (MC / GDP)*	6.8450 (5.1068)***	6.6376 (5.0239)***
*F*-statistic	77.6267	*ln (FC / GDP)*	-15.0970 (-9.1887)***	-14.8560 (-9.2358)***
Prob (*F*-statistic)	0.0000	*MC*	-4.74E-13 (-6.6461)***	-3.25E-13 (-4.7177)***
SD dependent var	2.5953	*constant*	106.0159 (16.8938)***	105.9788 (17.1923)***
		Adjusted *R*-squared	0.7404	0.7190
		SE of regression	11.8269	11.7251
		*F*-statistic	56.9857	51.2230
		Prob (*F*-statistic)	0.0000	0.0000
**Panel B: The effects of uncertainty avoidance (*UAI*) and status quo bias (*SQB*) on investment abroad concentration (*IAC*)**				
	***UAI*-*IAC_SQB***		***UAI-IAC_SQB*-*IAC***	
	**(48)**		**(49) *IAC***	**(50) *AL_IAC***
*UAI*	0.0007 (2.1196)**	*IAC_SQB*	0.3228 (10.7133)***	2.0824 (11.5802)***
*perGDP*	3.99E-09 (2.6560)***	*UAI*	0.0028 (9.8597)***	0.0190 (9.5394)***
*constant*	0.5728 (23.1968)***	*perGDP*	-5.90E-09 (-6.8580)***	-4.02E-08 (-7.9589)***
*R*-squared	0.0121	*GDP growth*	-0.0024 (-1.4455)	-0.0124 (-1.2457)
Adjusted *R*-squared	0.0101	*ln (ST / GDP)*	-0.0225 (-3.3498)***	-0.1428 (-3.5383)***
SE of regression	0.2571	*ln (MC / GDP)*	0.1001 (10.769)***	0.7238 (11.9404)***
*F*-statistic	6.1306	*ln (FC / GDP)*	-0.1841 (-13.248)***	-1.4968 (-17.5039)***
Prob (*F*-statistic)	0.0023	*MC*	-3.04E-15 (-5.7905)***	-4.08E-14 (-9.4223)***
SD dependent var	0.2584	*constant*	-0.3178 (-7.3019)***	-0.7751 (-3.1132)***
		Adjusted *R*-squared	0.8684	0.9079
		SE of regression	0.0903	0.6023
		*F*-statistic	161.0779	240.0830
		Prob (*F*-statistic)	0.0000	0.0000

Note: This table denotes the estimation results of uncertainty avoidance mediating impacts on the home bias (*HB*) and investment abroad concentration (*IAC*) according to Eqs (8) and (9). Yearly data are employed for the period 2001 to 2018. GLS with cross-section weights and the PCSE method are employed to perform the results. *SQB* denotes the status quo bias of investors. Explanatory and control variables include the *UAI* (culture of uncertainty avoidance), *perGDP* (GPD per capita), *GDP growth* (GDP growth rate), *ln (ST / GDP)* (the logarithm of stock traded to GDP), *ln (MC / GDP)* (the logarithm of the market capitalization of listed companies to GDP), *ln (FC / GDP)* (the logarithm of domestic credit provided by the financial sector to GDP), *MC* (market capitalization of listed companies).

*, **, *** indicate significance at the 10%, 5% and 1% levels, respectively.

Hypothesis 5, stating that investors’ status quo bias mediates the effect of uncertainty avoidance on investment underdiversification, is confirmed. Models 45 and 48 reveal that uncertainty avoidance has an effect on the degree of investors’ status quo bias of investors, which is in line with previous research. Investors are reluctant to change their established investment decisions due to ambiguity aversion [64–66]. Models 46 and 49 reveal that an increase in investors’ status quo bias leads to an increase in investment underdiversification, suggesting that investors with a high degree of status quo bias are satisfied with the current investment decisions and are not willing to invest in new overseas markets, which is consistent with the previous discussion.

In addition, this paper performs bootstrap tests to confirm the existence of the mediational effect of investors’ status quo bias. The maximum number of iterations is set as 3000, and the results are reported in Table 9. As the values of indirect and direct effects differ significantly from 0 and lower bounds of the 95% confidence intervals are all also greater than 0, it can be concluded that investors’ status quo bias has a mediational effect on the relationship between uncertainty avoidance and investment underdiversification.

**Table 9:** The results of bootstrap tests.

	Effect value	**SE.**	**LLCI**	**ULCI**
**Effect of explanatory variables (*UAI*) on mediator variables (*SQB*):**				
***HB***	0.0014***	0.0005	0.0014	0.0014
***IAC***	0.0016***	0.0003	0.0016	0.0016
**Effect of mediator variables (*SQB***.**) on explained variables (*UNDV*):**				
***HB***	0.8038***	0.2409	0.8037	0.8040
***IAC***	0.3355***	0.0594	0.3355	0.3356
**Direct effect of explanatory variables (*UAI*) on explained variables (*UNDV*):**				
***HB***	0.0073***	0.0057	0.0073	0.0073
***IAC***	0.0039***	0.0007	0.0033	0.0033

Note: This table denotes the bootstrap test results of uncertainty avoidance (*UAI*) on the home bias (*HB*) and investment abroad concentration (*IAC*). The maximum number of iterations is set as 3000.

## 5. Conclusions and implications

This study explores the effect and internal mechanism of uncertainty avoidance on investment underdiversification. Uncertainty avoidance is proxied by Hofstede’s cultural score of uncertainty avoidance index (*UAI*), and the degree of investment underdiversification in terms of overinvestment in a home country and overconcentration of investments abroad is measured by the Home Bias (*HB*) and Investment Abroad Concentration (*IAC*) indexes, respectively. The proposed models also consider economic and financial development control variables that may influence investors’ behavioral biases. The empirical results reveal that uncertainty avoidance is positively correlated to investment underdiversification, suggesting that the investments of investors from countries with high uncertainty avoidance are more underdiversified than those of investors from countries with low uncertainty avoidance. The robustness of these findings is confirmed by employing alternative variables and testing for endogeneity with the instrumental variable approach. A further analysis discovers that uncertainty avoidance moderates the effects of ambiguity on investment underdiversification, whereas the effect of uncertainty avoidance on investment underdiversification is mediated by investors’ degree of status quo bias.

These empirical findings contribute to existing research on the role of psychological traits in investment behavior and are of great importance for both researchers and financial market participants. Researchers should consider the effect of cultural background on investors’ psychological traits when analyzing cross-border financial activities and cross-border differences in economic and financial conditions, as culture is an important explanatory variable of investors’ behavior bias that is often overlooked in financial research. Financial market participants, such as fund managers, should consider clients’ cultural backgrounds and their impact on investment behavioral biases when constructing investment products to offer clients more appropriate portfolios that better meet the needs of investors with a certain degree of uncertainty avoidance.

Although our results offer a unique insight into a key channel through which proportions of national culture affect investment underdiversification, this research has several limitations and corresponding suggestions for future research orientation. Among all the cultural dimensions, only the culture of uncertainty avoidance is researched with the investment underdiversification in this study, while more cultural dimensions deserve future attention from financial culture researchers, such as individualism and long-term tendency. Moreover, with the updating of the Hofstede survey, future researchers can increase the coverage of more developing countries or regions to increase the generalisability of the finding.
